# The relationship of acute delirium with cognitive and psychiatric symptoms after stroke: a longitudinal study

**DOI:** 10.1186/s12883-022-02756-5

**Published:** 2022-06-27

**Authors:** Vilde Nerdal, Elise Gjestad, Ingvild Saltvedt, Ragnhild Munthe-Kaas, Hege Ihle-Hansen, Truls Ryum, Stian Lydersen, Ramune Grambaite

**Affiliations:** 1 Department of Psychology, Norwegian University of Science and Technology, Dragvoll Bygg 12, Edvard Bulls veg 1, 7491 Trondheim, Norway; 2 Clinic of Medicine, St. Olavs Hospital, Trondheim University Hospital, Trondheim, Norway; 3 Department of Neuromedicine and Movement Science, Norwegian University of Science and Technology, Trondheim, Norway; 4 Department of Geriatrics, St. Olavs Hospital, Trondheim University Hospital, Trondheim, Norway; 5 Department of Medicine, Bærum Hospital, Vestre Viken Hospital Trust, Drammen, Norway; 6 Institute of Clinical Medicine, University of Oslo, Oslo, Norway; 7 Department of Neurology, Oslo University Hospital, Oslo, Norway; 8 Department of Medicine, Bærum Hospital, Vestre Viken Hospital Trust, Sandvika, Norway; 9 Department of Mental Health, Faculty of Medicine and Health Sciences, Norwegian University of Science and Technology, Trondheim, Norway; 10 Health Services Research Unit (HØKH), Akershus University Hospital, Lørenskog, Norway

**Keywords:** Mental status and dementia tests, Cognitive dysfunction, Anxiety, Depression, Cerebrovascular event, Confusion

## Abstract

**Objective:**

Delirium, a common complication after stroke, is often overlooked, and long-term consequences are poorly understood. This study aims to explore whether delirium in the acute phase of stroke predicts cognitive and psychiatric symptoms three, 18 and 36 months later.

**Method:**

As part of the Norwegian Cognitive Impairment After Stroke Study (Nor-COAST), 139 hospitalized stroke patients (49% women, mean (*SD*) age: 71.4 (13.4) years; mean (*SD*) National Institutes of Health Stroke Scale (NIHSS) 3.0 (4.0)) were screened for delirium with the Confusion Assessment Method (CAM). Global cognition was measured with the Montreal Cognitive Assessment (MoCA), while psychiatric symptoms were measured using the Hospital Anxiety and Depression Scale (HADS) and the Neuropsychiatric Inventory-Questionnaire (NPI-Q). Data was analyzed using mixed-model linear regression, adjusting for age, gender, education, NIHSS score at baseline and premorbid dementia.

**Results:**

Thirteen patients met the criteria for delirium. Patients with delirium had lower MoCA scores compared to non-delirious patients, with the largest between-group difference found at 18 months (Mean (*SE*): 20.8 (1.4) versus (25.1 (0.4)). Delirium was associated with higher NPI-Q scores at 3 months (Mean (*SE*): 2.4 (0.6) versus 0.8 (0.1)), and higher HADS anxiety scores at 18 and 36 months, with the largest difference found at 36 months (Mean (*SE*): 6.2 (1.3) versus 2.2 (0.3)).

**Conclusions:**

Suffering a delirium in the acute phase of stroke predicted more cognitive and psychiatric symptoms at follow-up, compared to non-delirious patients. Preventing and treating delirium may be important for decreasing the burden of post-stroke disability.

**Supplementary Information:**

The online version contains supplementary material available at 10.1186/s12883-022-02756-5.

Stroke is ranked the third largest contributor to death and disability adjusted life years worldwide [1]. Cognitive impairment and psychiatric symptoms are prevalent in both the acute and chronic phases of stroke [2, 3]. These symptoms can interfere with restoration of daily function and independent living [4], placing emotional and economical burdens on patients, their families, and society [5, 6]. Identifying risk factors for post-stroke cognitive impairment and psychiatric symptoms is crucial for correct preventive measures and treatment.

The first 7 days of stroke, often referred to the acute phase [7], has key implications for long-term outcomes [8, 9], and complications during this phase has been shown to increase the risk of post-stroke sequelae [10]. Delirium is a common complication in acute stroke and has been suggested as a potential risk factor for later dependency and dementia [2, 11]. The Diagnostic and Statistical Manual of Mental Disorders, 5th edition (DSM-5), defines delirium as an acute and fluctuating disturbance of attention, cognition and/or consciousness, which occurs due to medical conditions and cannot be better explained by a pre-existing neurocognitive disorder [12]. Studies have found a prevalence of delirium in the acute phase of stroke ranging from 8 to 48% [13]. A Norwegian stroke unit found that 10% of stroke patients had delirium [14].

Delirium often occurs as a response to brain injury [15], such as stroke [16], but the etiology is poorly understood. The lack of knowledge of causal mechanisms of delirium, combined with its fluctuating and heterogeneous nature, can challenge detection of the condition. The gold standard for diagnosing delirium is a clinical evaluation by professionals using the DSM-5 criteria [17]. However, briefer screening tools are usually more feasible in acute settings. The Confusion Assessment Method (CAM) [18] is a screening tool found noninferior to diagnosis made by trained neurologists using the DSM-5 criteria, with sensitivity of 76% and specificity of 98% [19].

Experiencing delirium can be stressful and emotionally challenging for the patient [20]. Stroke patients with delirium have higher mortality rates and longer hospital stays [21]. Furthermore, the condition increases the risk of falls and hospital acquired infections [22]. It predisposes for worse functional outcomes [23, 24] and less functional independence by the time of discharge [21].

Studies have found delirium to be associated with post-stroke cognitive impairment for up to 2 years after stroke [25]. However, the literature is inconsistent, and other studies have found the negative effect of delirium diminishing after 3 months [26] and after 12 months [27]. As neither of the previous studies adjusted for pre-stroke dementia or stroke severity, the effect of delirium on cognition and psychiatric symptoms is left somewhat unclear.

Fleischmann et al. [17] theorizes that delirium may hinder the standard course of stroke rehabilitation, not just by delaying physical training, but also by interfering with cognitive interventions. This further highlights the relevance of examining whether the condition is associated with poorer long-term cognitive outcomes.

Several of the clinical consequences of delirium, such as longer hospitalization and poorer functional outcomes, are also associated with increased risk of developing psychiatric symptoms, such as depression [28, 29]. However, recent studies have mainly addressed post-stroke depression in general stroke populations, leaving the impacts of delirium largely unexplored. Further, there is an even greater absence of research on how delirium is linked to a broader range of psychiatric symptoms, such as anxiety, which often overlaps with depressive symptoms [30]. This study aims to explore whether delirium in the acute phase of stroke predicts global cognitive function, as well as symptoms of depression, anxiety, and general psychiatric distress over the course of 3 years.

## Method

### Participants

The present sub-study is a part of the Norwegian Cognitive Impairment After Stroke Study (Nor-COAST). Nor-COAST is a longitudinal multicenter, prospective cohort study that recruited 815 participants from five Norwegian hospitals from May 2015 to March 2017 [31]. The inclusion criteria were diagnosis of acute stroke, admittance to hospital within 7 days of symptom onset, age over 18 years, and fluency in a Scandinavian language. Stroke was diagnosed according to the World Health Organization criteria or with findings of acute intra-cerebral hemorrhage or infarction on Magnetic Resonance Imaging (MRI). The only exclusion criterion was a life expectancy of less than 3 months.

Our sub-study included only patients from Bærum Hospital, Vestre Viken Health Trust, excluding patients from the other four hospitals. This hospital implemented a consistent regime for delirium screening, ensuring that all patients were screened regularly during the first 2 days after admittance.

Aphasia was not an exclusion criterion in the Nor-COAST study, nor in this particular study. Patients with aphasia were however automatically excluded from MoCA assessments by research nurses, but not for HADS or NPI-Q assessments.

### Premorbid function and health history

Health history was collected from medical records, and interviews with either the patient or the caregiver were used to register premorbid function, including The Global Deterioration Scale (GDS) [32] and the Charlson Comorbidity Index [33]. The Global Deterioration Scale (GDS) [32] was used to assess cognitive function before the stroke, and after three, 18 and 36 months. Pre-stroke GDS was assessed by the local principal investigator at inclusion based on interview of the patients, the proxy and medical records. GDS has seven stages, where 4–7 are considered dementia stages and 1–3 are considered pre-dementia stages, with stage 3 being equivalent to mild cognitive impairment (MCI) [34]. The Charlson Comorbidity Index [33] was used to classify the extent of comorbid diseases as mild (0–2), moderate [3–5] or severe (≥5), and was registered upon admission.

### Stroke characteristics and complications

Stroke severity was measured using the National Institutes of Health Stroke Scale (NIHSS) [35] at day one of hospitalization. NIHSS has 15 items measuring specific symptoms on a 3- or 4-point ordinal scale (0 = no impairment). The highest score possible for non-comatose patients is 42. The NIHSS item measuring language difficulties was also used to identify patients with moderate and severe aphasia (i.e., causing interference with conversation). Further, complications during stroke were defined as the number of infections, falls, progression in stroke symptoms and seizures during hospitalization, which were then pooled into a sum score and recorded upon discharge.

### Delirium

Delirium was diagnosed using CAM [18]. Screenings were performed by nurses specialized in stroke care once during every shift during the first 48 hours of hospitalization, resulting in a total of six screenings per patient. The majority of patients were screened at fixed intervals (7:00, 15:00 and 21:00). Delirium is defined based on four features in CAM: 1) the acute onset of fluctuations or changes in the mental status of the patient, 2) inattention, 3) disorganized thinking, and 4) an altered level of consciousness. To increase the sensitivity of the inattention item, a task was added, asking the patients to name the days of the week backward [36–38]. The diagnosis of delirium required the presence of feature 1 and 2, plus feature 3 or 4. Feature 2 was considered present either if indicated by the inattention item in CAM, or if the patient named less than 7 days backwards and aphasia was not indicated. Screening with CAM was integrated in the clinical observations of the patients. The patient’s families were not involved in the CAM screening. Pre-stroke GDS-score and study notes on pre-stroke history provided a reference point for the patient’s normal behavior.

### Cognitive outcome

Global cognitive function was measured using the Montreal Cognitive Assessment (MoCA) [39]. MoCA assesses several cognitive domains including executive function, memory, language, visuospatial abilities, attention and working memory. The maximum score is 30, with higher scores indicating better cognition. Scores of 26 and above are considered normal cognitive function [39]. Patients were tested at baseline and after three, 18 and 36 months. Baseline assessment of cognitive function was performed either at discharge, or at day seven for patients with longer stays. Follow-up assessments were performed by research nurses and physicians specializing in stroke at an outpatient clinic. If the patients were unable to attend the clinic, follow-up was completed by telephone.

### Psychiatric outcomes

The Hospital Anxiety and Depression Scale (HADS) [40] was used to assess symptoms of anxiety and depression. HADS consists of 14 items assessing two subscales (anxiety: HADS-A and depression: HADS-D). The items are rated on a 4-point scale from 0 (not at all) to 3 (most of the time), and a score of 8 or more on either subscale may indicate clinically relevant symptoms [41]. The Neuropsychiatric Inventory-Questionnaire (NPI-Q) [42] was used to assess symptoms according to 12 neuropsychiatric domains, such as delusions, hallucinations, agitation, motor disturbance and aggression. The score for each domain ranged from 0 (no symptoms) to 4 (severe symptoms), with the total score reflecting the sum of the individual domains. Both NPI-Q and HADS were assessed at three, 18 and 36 months post-stroke.

### Statistics

A statistical analysis plan was completed prior to conducting the analyses. MoCA sum scores were used to analyze cognitive symptoms, while sum scores from HADS-A, HADS-D and NPI-Q were used to analyze psychiatric symptoms. Imputation of the mean value of missing items was performed if ≥50% of the items had data [43] for MoCA, HADS and NPI-Q. The number of missing data on single items was low. Details are shown in Supplementary Table S1.

Normality of residuals was checked by visual inspection of Q-Q plots. Differences between groups (delirium vs. non-delirious) were analyzed using t-tests for continuous variables, and chi-square test for categorical variables. This was conducted to explore whether the results from the Bærum sample would be representative for the total Nor-COAST sample. In cases with expected count under 5, we used the unconditional z-pooled test as recommended by Lydersen, Langaas and Bakke [44].

Mixed-model linear regression was applied with MoCA, HADS and NPI-Q one at a time, as dependent variables. Independent variables were delirium, time as a categorical covariate and their interaction. The analyses were adjusted for age, gender, education, NIHSS score at baseline and premorbid dementia. Premorbid dementia was defined as being diagnosed with dementia, premorbid GDS score over 3 and/or premorbid usage of anti-dementia medication and/or other previous treatment for dementia.

Linear mixed-models includes and analyzes participants with available data on at least one time point, and give unbiased estimated if data are missing at random [45]. Sensitivity analyses (excluding patients deceased at 18 and/or 36 months and patients with premorbid dementia) were conducted, as well as analyses adjusted for comorbidity and complications. Finally, analyses excluding outliers and analyses excluding patients with moderate and severe aphasia were conducted for HADS and NPI-Q.

In this paper, the term “significant” refers to two tailed *p*-values less than .05, and 95% confidence intervals (CIs) are reported where relevant. SPSS 27 was used for the analyses.

## Results

### Participants

**Fig. 1:**
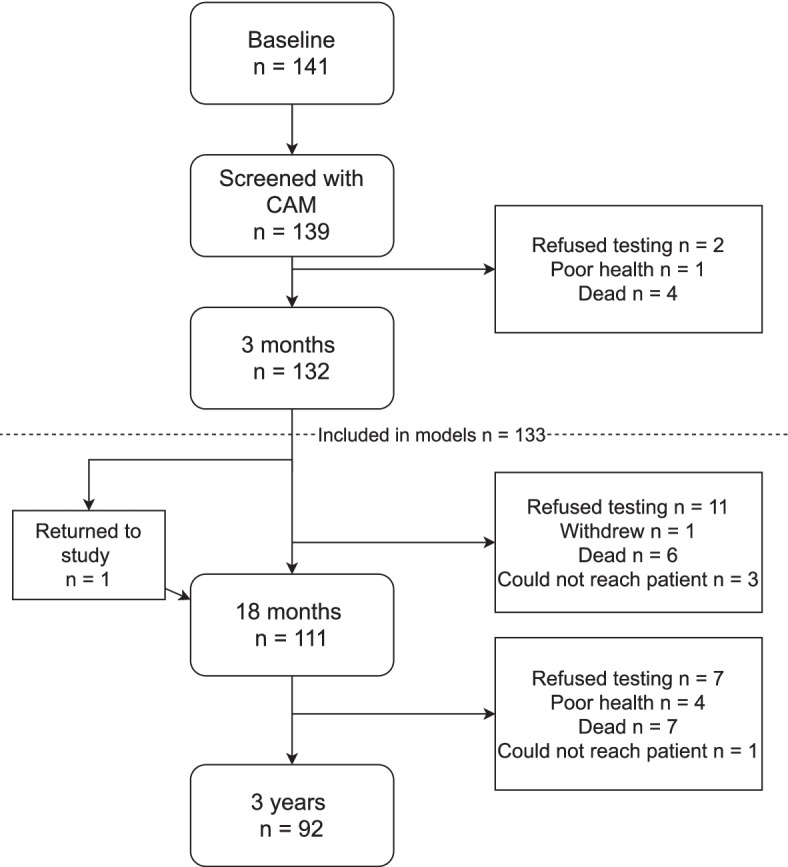
Overview of the study sample. CAM (Inouye, 1994) was used to screen for delirium. Each patient was screened six times during the first 48 hours. Delirium was considered present if the patients had acute onset of fluctuations in mental status and inattention, in addition to disorganized thinking and/or altered level of consciousness at one or more of the screenings. Patients were included in mixed models if they had been screened at baseline and returned for at least one additional follow-up

As shown in Fig. 1, 139 of the 141 patients included in the Nor-COAST study from Bærum Hospital, were screened for delirium. Of these patients, 133 had the data necessary to be included in the mixed-model linear regression analyses.

At 3 months, 132 patients attended the follow-up. At 18 months, 111 patients participated, one of which did not participate at the previous follow-up. At 36 months, 92 patients participated in the follow-up. Compared to the four other hospitals participating in the Nor-COAST study (St. Olavs Hospital, Haukeland Hospital, Ullevål Hospital and Ålesund Hospital), patients at Bærum Hospital were significantly younger, had more years of education, higher MoCA scores at baseline, less pre-stroke dementia, less comorbidity and lower pre-stroke GDS score (Table 1).

**Table 1:** Group differences in demographics and clinical characteristics for Bærum Hospital and all other hospitals

	Patients at Bærum Hospital (*n* = 141)	Patients from all other hospitals (*n* = 674)	*t/ x*^*2*^	*p*
Age, *M* (*SD*)	71.4 (13.4)	74.0 (11.3)	2.21	.02*
Years of education, *M* (*SD*)	13.9 (3.5)	11.5 (3.6)	−6.9	.000**
Gender, n female (%)	68 (49%)	302 (36%)	−.03	.75
NIHSS at baseline^a^, *M* (*SD*)	3.0 (4.0)	3.67 (5.04)	.78	.45
MoCA at baseline^b^, *M* (*SD*)	24.7 (4.6)	22.6 (5.1)	−4.6	.000**
Premorbid dementia, n (%)	2 (1.4%)	63 (9.5%)	10.1	.001**
Complications^c^ > 0, n (%)	34 (24.3%)	160 (25%)	.04	.85
Charlson Comorbidity Index (CCI), *M (SD)*	3.6 (1.8)	4.2 (2.0)	3.4	.001**
Pre-stroke Global Deterioration Scale <3^d^, n (%)	138 (98.6%)	554 (80%)	27.8	.000**
Intracerebral hemorrhage, n (%)	16 (11.5%)	62 (9.2%)	0.6	.42
Arterial ischemic stroke, n (%)	120 (86.1%)	543 (81.6%)	2.1	.14

^a^Higher values indicating more severe stroke symptoms. NIHSS at baseline assessed at day 1 of admittance to hospital

^b^Lower values indicating poorer global cognitive function. MoCA assessment at baseline was performed either at discharge or 7 days after admittance for patients with longer hospital stay

^c^Infections, seizures, neurological progression and falls registered during hospitalization

^d^Values < 3 indication no to very mild cognitive decline

**indicating *p*-level < .01

*indicating *p*-level < .05

Of the 139 screened with CAM, 13 (9.4%) were diagnosed with delirium. Demographics and clinical characteristics of patients with delirium and non-delirious patients are shown in Table 2. Patients with delirium were older and had lower MoCA score at baseline. Further, a higher percentage of patients with delirium had GDS scores > 3 (in the dementia range) at three, 18 and 36 months (Table 2). Patients were hospitalized for mean 0.84 days, and 88% were admitted within day one of symptom debut.

**Table 2:** Group differences in demographics and clinical characteristics for patients with and without delirium

	Delirium (*n* = 13)	Non-delirious (*n* = 126)	*t/ x*^*2*^	*p*
Age, *M* (*SD*)	79.5 (6.0)	70.6 (13.7)	4.34	.000**
Years of education, *M* (*SD*)	12.5 (3.5)	13.9 (3.4)	−1.43	.15
Gender, n female (%)	6 (46%)	62 (49%)	.04	.84
NIHSS at basline^a^, *M* (*SD)*	4.5 (4.6)	2.8 (3.8)	1.35	.17
MoCA at baseline^b^, *M* (*SD*)	20.0 (2.2)	25.1 (4.7)	−3.10	.002**
Premorbid dementia, n (%)	0	2	.19	.91
Complications^c^ > 0, n (%)	7 (54.6)	27 (21.4)	−2.15	.05
Charlson Comorbidity Index (CCI), *M (SD)*	4.1 (1.3)	3.6 (1.9)	−1.20	.24
Global Deterioration Scale (GDS) < 3^d^				
Pre-stroke, n (%)	12 (92%)	124 (98%)	.19	.66
3 months, n (%)	4 (30%)	5 (4%)	14.0	.003**
18 months, n (%)	4 (31%)	11 (9%)	5.9	.024*
36 months, n (%)	3 (23%)	10 (8%)	3.2	.079
Moderate to severe aphasia^e^				
Baseline, n (%)	3 (23%)	19 (15%)	.57	0.57
3 months, n (%)	0	4 (3%)	.42	0.68
18 months, n (%)	0	3 (2%)	.32	0.81
36 months, n (%)	0	0	0	1.0

^a^Higher values indicating more severe stroke symptoms. NIHSS at baseline done at day 1 of admittance to hospital

^b^Lower values indicating poorer global cognitive function. MoCA assessment at baseline was done either at discharge or 7 days after admittance for patients with longer hospital stay

^c^Infections, seizures, neurological progression and falls registered during hospitalization

^d^Values < 3 indication no to very mild cognitive decline. Values > 3 indicating potential dementia

^e^Amount of patients with a level of aphasia causing interference with conversation, indicated by the value 2 (moderate) or 3 (severe) in the NIHSS item measuring aphasia

**indicating *p*-level < .01

*indicating *p*-level < .05

### Delirium as a predictor for cognitive outcomes

**Fig. 2:**
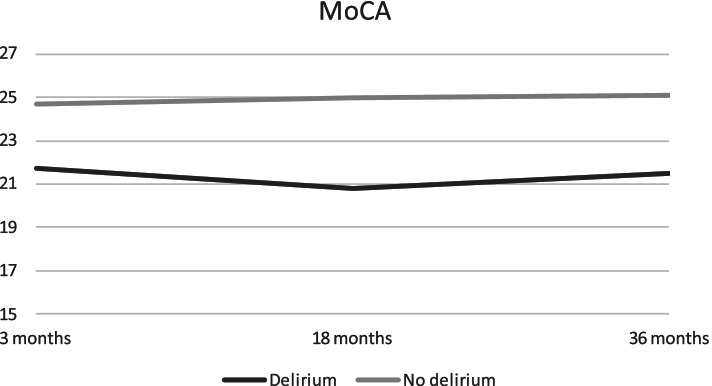
MoCA total scores for patients with and without delirium at three, 18 and 36 months. Descriptive mean total-scores for MoCA after three, 18 and 36 months for patients from Bærum with and without delirium

Cognitive assessments for patients with and without delirium are described in Table 3 and displayed in Fig. 2. Delirium at baseline predicted significantly lower MoCA score at all timepoints. The largest difference in mean scores between the two groups was 4.2 points (95% CI: 1.4 to 7.1) at 18 months. The results were substantially the same in sensitivity analyses excluding patients with premorbid dementia, patients that were deceased at 18 and/or 36 months, and when adjusting complications, and comorbid diseases (results not shown). The variance components in the mixed models are shown in Table 4.

**Table 3:** Assessments for patients at Bærum Hospital with and without delirium during first 48 hours

	Delirium *n*	Mean (*SE*)	Non-delirious *n*	Mean (*SE*)	Difference Estimate (95% CI)	*p*
**3 months**						
MoCA	10	21.7 (1.4)	115	24.7 (0.4)	2.9 (0.1 to 5.7)	.04*
NPIQ	10	2.4 (0.6)	117	0.8 (0.1)	−1.6 (−2.7 to −0.47)	.005**
HADS Depression	8	3.7 (1.3)	100	3.7 (0.3)	0.04 (−2.6 to − 2.7)	.97
HADS Anxiety	8	4.0 (1.1)	100	3.1 (0.3)	- 0.9 (−3.4 to 1.4)	.44
**18 months**						
MoCA	9	20.8 (1.4)	100	25.0 (0.4)	4.2 (1.4 to 7.1)	.004*
NPIQ	9	0.6 (0.6)	100	1.0 (0.2)	0.44 (− 0.8 to 1.7)	47
HADS Depression	7	6.2 (1.4)	93	3.7 (0.3)	- 2.44 (− 5.2 to 0.3)	.08
HADS Anxiety	7	5.6 (1.2)	93	3.1 (0.3)	- 2.6 (−5.0 to 0.1)	.04*
**36 months**						
MoCA	5	21.5 (1.5)	83	25.1 (0.4)	3.6 (0.5 to 6.7)	.02*
NPIQ	5	1.7 (0.7)	84	0.7 (0.2)	−1.0 (− 2.5 to 0.5)	.17
HADS Depression	5	6.3 (1.4)	75	4.1 (0.4)	- 2.3 (− 5.1 to 0.64)	.12
HADS Anxiety	5	6.2 (1.3)	75	3.3 (0.3)	- 2.8 (− 5.5 to - 0.3)	.03*

Mean (*SE*) are descriptive data. The difference with CI and *p*-value are from linear mixed models with covariates delirium, time and their interaction as categorical covariates, adjusted for age, gender, NIHSS-score at baseline and premorbid dementia.

* Indicating *p* < .05 and ** indicating *p* < .01

**Table 4:** Variance components in linear mixed model reported in Table 3

	Variance Component	
Dependent variable	Within participants	Between participants
MoCA	13.38 (1.7)	3.7 (.38)
NPI-Q	1.8 (.18)	1.0 (.24)
HADS Depression	4.16 (.46)	8.4 (1.4)
HADS Anxiety	3.76 (.41)	6.1 (1.0)

Variance components are from linear mixed models with delirium, time and their interaction as categorical covariates, adjusted for age, gender, NIHSS-score at baseline and premorbid dementia

### Delirium as a predictor for psychiatric outcomes

**Fig. 3:**
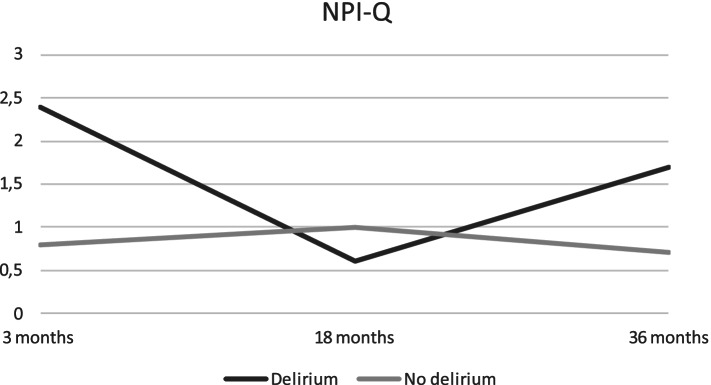
NPI-Q total scores for patients with and without delirium at three, 18 and 36 months. Descriptive mean total-scores for NPI-Q after three, 18 and 36 months for patients from Bærum with and without delirium

**Fig. 4:**
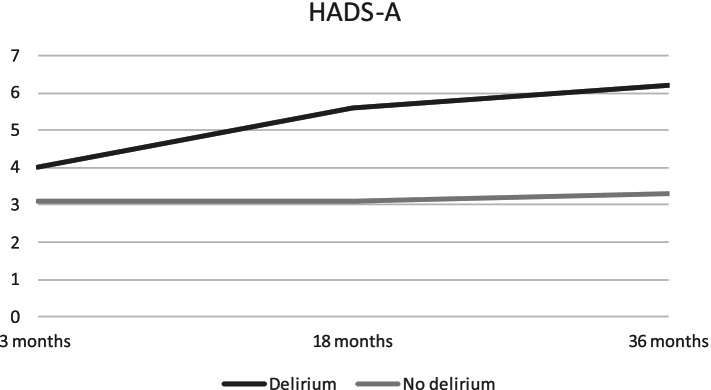
HADS-D scores for patients with and without delirium at three, 18 and 36 months. Descriptive mean for HADS-A after three, 18 and 36 months for patients from Bærum with and without delirium

**Fig. 5:**
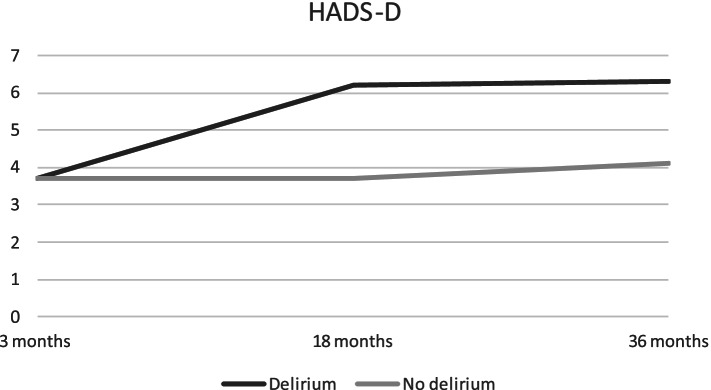
HADS-A scores for patients with and without delirium at three, 18 and 36 months. Descriptive mean scores for HADS-D after three, 18 and 36 months for patients from Bærum with and without delirium

Delirium predicted significantly higher NPI-Q scores, compared to non-delirious patients after 3 months (Table 3). Anxiety symptoms measured by HADS-A increased continuously for patients with delirium, resulting in significantly higher anxiety scores at 18 and 36 months, compared to non-delirious patients. The largest difference in mean scores for anxiety between the two groups was found at 36 months (2.8 points (95% CI: − 5.5 to − 0.3)). Mean scores for NPI-Q, HADS-A and HADS-D are displayed in Figs. 3, 4 and 5 respectively.

## Discussion

This study examined whether delirium in the acute phase of stroke predicts cognitive and psychiatric symptoms over the course of 3 years. We demonstrated that patients with delirium had significantly poorer global cognition (MoCA scores) than non-delirious patients at three, 18 and 36 months. Delirium also predicted significantly higher levels of neuropsychiatric symptoms (NPI-Q) after 3 months, as well as higher anxiety levels (HADS-A) at 18 and 36 months.

MoCA is estimated to have a sensitivity of 90% for detecting MCI using a cut-off score of 26 [39]. This is in line with a previous publication from the Nor-COAST study by Munthe-Kaas et al. [46], who found that a MoCA cut-off of 26 had high specificity and sensitivity for identifying patients with cognitive impairment (according to the DSM-5 criteria) 3 months post-stroke. Further, Nasreddine et al. [39] suggest that MoCA scores in the range of 11.4–21.0 indicate potential dementia. These estimations suggest that the average MoCA score of stroke patients with delirium in this study (21.5 at 36 months) could indicate more severe clinical outcomes, compared to the MoCA scores of non-delirious patients (25.1 at 36 months).

Several studies have reported delirium as a risk factor for cognitive impairment in hospitalized patients [36, 47–50], and in patients with stroke [25–27]. However, the independent effect of delirium on post-stroke cognitive impairment remains somewhat unexplored, as few studies have adjusted for other risk factors such as stroke severity, pre-stroke dementia, complications, and comorbid diseases. In this study, the patients with delirium had fewer years of education, more complications, more comorbid diseases, a higher mean NIHSS score, and were significantly older, than the non-delirious patients. It should be stressed that the association between delirium and poorer MoCA scores remained significant even when adjusting for these covariates in the sensitivity analyses, suggesting an independent effect of delirium on global cognition. Further, these results were found despite the Bærum sample having several more protective factors [51], such as lower age, longer education, and milder strokes, compared to the total Nor-COAST sample (Table 1). This raises a question as to whether delirium would predict more severe cognitive impairment in older samples with larger strokes.

The results for HADS and NPI-Q remained significant when adjusting for aphasia. This could be due to the majority of patients having mild strokes and the rates of aphasia being low. Additionally, we only adjusted for moderate and severe aphasia (i.e., interfering with conversation). In future studies it would be beneficial to adjust for mild aphasia and explore the effect of aphasia and other complications in samples with more severe strokes.

Although patients with delirium had higher HADS-A scores than non-delirious patients in this study, the average score was not above the commonly used cut-off score of 8 [41]. However, adjusting HADS cut-off scores to the specific clinical sample can provide a higher specificity and sensitivity [52, 53]. Sagen et al. [53] found a cut-off score of 4 to be optimal in Norwegian stroke populations for detecting clinical symptoms. This would imply that the anxiety levels in patients with delirium are of clinical importance at 18 and 36 months (Mean (*SD*): 5.6 (1.2) and 6.2 (1.3) respectively). Though Kowalska et al. [54] recently found delirium to be a risk factor for anxiety 3 months after stroke, our findings suggest that the subsequent anxiety symptoms can be present or even increase over a longer timeframe.

In the present study, both HADS-A and HADS-D scores were higher in patients with delirium compared to non-delirious patients. However, group differences were only significant for HADS-A. This corresponds to several other Norwegian stroke studies, finding anxiety symptoms to be more prevalent than depressive symptoms using HADS [53, 55, 56]. However, a meta-analysis from 2017 found higher prevalence of post-stroke depressive disorders than post-stroke anxiety (33.5% versus 9.8%), using DSM or World Health Organization criteria (ICD-10) [57]. A potential explanation for this discrepancy is that anxiety is more commonly overlooked in older samples, as symptoms like withdrawal and avoidant coping strategies can be falsely attributed to aging [58]. This could lead to an underestimation of anxiety symptoms in stroke populations.

MacLullich, Beaglehole, Hall & Meagher [59] emphasizes stress as a potential mediator between delirium and anxiety symptoms. Systems related to the physiological stress response, such as the limbic hypothalamic-pituitary-adrenal axis (HPA axis) and glucocorticoids, have been found to precipitate and/or sustain delirium both in acute disease [60, 61] and in stroke specifically [62]. Intense or prolonged stress responses can initiate increased vigilance and symptoms of anxiety [63, 64], and several studies address dysregulations of the HPA axis and glucocorticoid production as central for this association [64–66]. Further, older age is associated with increased dysregulations of the HPA axis, causing cortisol levels to be sustained for a longer period of time after major stressors [67, 68]. The Bærum sample had a lower mean age than the total Nor-COAST sample and the general stroke population in Norway [69]. Considering the age-related risk of HPA dysregulation, the association between delirium and anxiety may be even stronger in a larger and more representative population.

A limitation of this study was using CAM, rather than clinical evaluation, to assess for delirium. CAM has not been validated for stroke patients. The CAM-ICU [70] has however been evaluated as a valid instrument for diagnosing delirium in patients with stroke [71]. The CAM-ICU consist of the same four features as CAM (acute onset of changes or fluctuations in mental status; inattention; disorganized thinking and/or altered level of consciousness) [70, 72]. CAM-ICU is however adapted to mechanically ventilated patients with more severe strokes, as the questions are nonverbal. Our sample had mild strokes and low rates of aphasia, making the original CAM more relevant.

As delirium has a fluctuating and heterogenous nature, it can be easy to overlook or mislabel delirium on other cerebral dysfunction with similar expression. Detecting individual deviations in attention can be particularly challenging without any point of reference [38]. The decision to use data only from Bærum Hospital was made in an attempt to meet some of these challenges. Bærum Hospital performed highly consistent delirium screenings for each patient, increasing the chance of picking up these fluctuations [17]. The majority of patients at Bærum (88%) were admitted to hospital within day one of symptom debut. Almost all patients admitted with stroke were screened six times, regardless of symptoms or health history, and the prevalence rate at this hospital (9.6%) resembled that of another Norwegian study of delirium (10%) [14]. The screenings were done by nurses specialized in stroke care, and prior experience with delirium could assist in adequately identifying the condition. Further, the item added to CAM has been shown to increase the sensitivity for detecting disturbances in attention [38], and the nurses access’ to pre-stroke GDS and medical history provided a reference point for normal behavior. This might help increase the validity of the screening. Delirium developing later than 48 hours after stroke would not have been detected, but as the condition is most eminent in the acute phase of disease [15], most cases were likely identified.

The sample assessed in this study was relatively small, and the demographic differences between the Bærum sample and the total Nor-COAST sample could impact the representativity of the results. Further, most patients at Bærum hospital had mild strokes (see Table 1). This could lead to lower rates of delirium, aphasia, and other stroke-related complications, compared to samples with more severe strokes. However, Kuvås and colleagues [73] found the Nor-COAST sample to be representative for the majority of patients suffering from mild strokes, which is valuable as the majority of the Norwegian stroke population (64%) experience mild strokes [74]. Future studies should be conducted to examine the effect of delirium on cognition and psychiatric symptoms in a larger sample, maintaining a meticulous screening regime. Adjusting for stroke type, size, localization, white matter lesions, microbleeds, brain atrophy and volume loss could further be relevant, as these variables are found closely related to both cognitive [75] and psychiatric outcome [76] after stroke. Frailty, often used to express the accumulation of functional deficits [77], has been shown to be associated with cognitive impairment after stroke [78]. Therefore, it might also be relevant to examine delirium in stroke in relation to the concept of frailty.

Still, identifying an independent effect of delirium can pose some challenges, as the condition often evolves from the interaction of multiple pathological factors [79]. Nevertheless, delirium can be considered a crucial indicator for adequate measures to be taken. This perspective is relevant, as there are several non-pharmaceutical measures available that reduce the symptoms of delirium, such as frequently reorienting and touching the patient, providing a calendar and a watch, and having an unambiguous approach with sufficient eye contact [80]. However, acknowledging the short- and long-term burden of delirium can be of importance for these measures to be implemented in stroke units.

## Conclusion

Patients suffering from delirium in the acute phase of stroke had poorer global cognition and more psychiatric symptoms over the course of 3 years, compared to non-delirious patients. The results suggest that stroke patients with delirium may benefit from long-term follow-up of both cognition and mental health, and that prevention and treatment of delirium presents an interesting future approach. Distributing knowledge on the short- and long-term burden of delirium may be important for adequate measures to be taken.

## Supplementary Information

**Additional file 1: Table S1.** Details on imputation of missing values for MoCA, HADS and NPI-Q at all timepoints.

## Data Availability

The datasets generated and analyzed in this study are not publicly available due to privacy concerns but are available from the corresponding author on reasonable request.

## Abbreviations

Nor-COAST: Norwegian Cognitive Impairment After Stroke Study
NIHSS: National Institutes of Health Stroke Scale
CAM: Confusion Assessment Method
MoCA: Montreal Cognitive assessment Method
HADS: Hospital Anxiety and Depression Scale
HADS-A: HADS Anxiety
HADS-D: HADS Depression
NPI-Q: Neuropsychiatric Inventory Questionnaire
DSM-5: Diagnostic and Statistical Manual of Mental Disorders, 5th edition
CCI: Charlson Comorbidity Index
GDS: Global Deterioration Scale
MCI: Mild Cognitive Impairment
ICD-10: International Classification of Diseases
HPA-axis: (Hypothalamic-Pituitary-Adrenal axis)

## References

[1] Vos T, Lim SS, Abbafati C, Abbas KM, Abbasi M, Abbasifard M, et al. Global burden of 369 diseases and injuries in 204 countries and territories, 1990–2019: a systematic analysis for the Global Burden of Disease Study 2019. Lancet Neurol. 2020;396(10258):1204–22. 10.1016/S0140-6736(20)30925-9 PMC7567026 33069326

[2] Ferro JM, Caeiro L, Figueira ML. Neuropsychiatric sequelae of stroke. Nat Rev Neurol. 2016;12(5):269–80. 27063107 10.1038/nrneurol.2016.46

[3] Leys D, Henon H, Mackowiak-Cordoliani MA, Pasquier F. Poststroke dementia. Lancet Neurol. 2005;4(11):752–9. 16239182 10.1016/S1474-4422(05)70221-0

[4] Pohjasvaara T, Leskelä M, Vataja R, Kalska H, Ylikoski R, Hietanen M, et al. Post-stroke depression, executive dysfunction and functional outcome. Eur J Neurol. 2002;2002(9):269–75. 10.1046/j.1468-1331.2002.00396.x 11985635

[5] Johnson CO, Nguyen M, Roth GA, Nichols E, Feigin VL, Vos T, et al. Global, regional, and national burden of stroke, 1990–2016: a systematic analysis for the Global Burden of Disease Study 2016. Lancet Neurol. 2019;18(5):439–58. 30871944 10.1016/S1474-4422(19)30034-1 PMC6494974

[6] Rajsic S, Gothe H, Borba HH, Sroczynski G, Vujicic J, Toell T, et al. Economic burden of stroke: a systematic review on post-stroke care. Eur J Health Econ. 2019;20(1):107–34. 29909569 10.1007/s10198-018-0984-0

[7] Bernhardt J, Hayward KS, Kwakkel G, Ward NS, Wolf SL, Borschmann K, et al. Agreed definitions and a shared vision for new standards in stroke recovery research: The Stroke Recovery and Rehabilitation Roundtable taskforce. Int J Stroke. 2017;12(5):444–50. 28697708 10.1177/1747493017711816

[8] McDermott M, Jacobs T, Morgenstern L. Critical care in acute ischemic stroke. Handb Clin Neurol. 2017;140:153–76. 10.1016/B978-0-444-63600-3.00010-6. 10.1016/B978-0-444-63600-3.00010-6 28187798

[9] Pendlebury ST. Screening for Delirium in Acute Stroke. Stroke. 2021;52(1):479–81. 33380166 10.1161/STROKEAHA.120.033192

[10] Pandian JD, Kaur A, Jyotsna R, Sylaja PN, Vijaya P, Padma MV, et al. Complications in acute stroke in India (CAST-I): a multicenter study. J Stroke Cerebrovasc Dis. 2012;21(8):695–703. 21511495 10.1016/j.jstrokecerebrovasdis.2011.03.003

[11] Eeles EMP, Hubbard RE, White SV, O’Mahony MS, Savva GM, Bayer AJ. Hospital use, institutionalisation and mortality associated with delirium. Age Ageing. 2010;39(4):470–5. 20554540 10.1093/ageing/afq052

[12] American Psychiatric Association. Diagnostic and statistical manual of mental disorders. 5th ed. Arlington: American Psychiatric Association; 2013.

[13] Qu J, Chen Y, Luo G, Zhong H, Xiao W, Yin H. Delirium in the Acute Phase of Ischemic Stroke: Incidence, Risk Factors, and Effects on Functional Outcome. J Stroke Cerebrovasc Dis. 2018;27(10):2641–7. 30172676 10.1016/j.jstrokecerebrovasdis.2018.05.034

[14] Dahl MH, Rønning OM, Thommessen B. Delirium in acute stroke – prevalence and risk factors. Acta Neurol Scand. 2010;122(s190):39–43. 10.1111/j.1600-0404.2010.01374.x 20586734

[15] Caeiro L, Ferro JM, Albuquerque R, Figueira ML. Delirium in the first days of acute stroke. J Neurol. 2004;251(2):171–8. 14991351 10.1007/s00415-004-0294-6

[16] Ferro JM, Caeiro L, Verdelho A. Delirium in acute stroke. Curr Opin Neurol. 2002;15(1):51–5. 11796951 10.1097/00019052-200202000-00009

[17] Fleischmann R, Warwas S, Andrasch T, Kunz R, Witt C, Mengel A, et al. Course and Recognition of Poststroke Delirium. Stroke. 2021;52(2):471–8. 33380165 10.1161/STROKEAHA.120.031019

[18] Inouye SK, van Dyck CH, Alessi CA, Balkin S, Siegal AP, Horwitz RI. Clarifying confusion: the confusion assessment method. A new method for detection of delirium. Ann Intern Med. 1990;113(12):941–8. 2240918 10.7326/0003-4819-113-12-941

[19] Mansutti I, Saiani L, Palese A. Detecting delirium in patients with acute stroke: a systematic review of test accuracy. BMC Neurol. 2019;19(1):310. 31791260 10.1186/s12883-019-1547-4 PMC6889202

[20] Wilson JE, Mart MF, Cunningham C, Shehabi Y, Girard TD, MacLullich A, Slooter A, Ely EW. Delirium. Nature reviews. Dis Prim. 2020;6(1):90. 10.1038/s41572-020-00223-4. 10.1038/s41572-020-00223-4 PMC9012267 33184265

[21] Shi Q, Presutti R, Selchen D, Saposnik G. Delirium in Acute Stroke. Stroke. 2012;43(3):645–9. 22267831 10.1161/STROKEAHA.111.643726

[22] Pendlebury ST, Lovett NG, Smith SC, Dutta N, Bendon C, Lloyd-Lavery A, et al. Observational, longitudinal study of delirium in consecutive unselected acute medical admissions: age-specific rates and associated factors, mortality and re-admission. BMJ Open. 2015;5(11):e007808. 26576806 10.1136/bmjopen-2015-007808 PMC4654280

[23] Naidech AM, Beaumont JL, Rosenberg NF, Maas MB, Kosteva AR, Ault ML, et al. Intracerebral Hemorrhage and Delirium Symptoms. Length of Stay, Function, and Quality of Life in a 114-Patient Cohort. Am J Respir Crit Care Med. 2013;188(11):1331–7. 24102675 10.1164/rccm.201307-1256OC PMC3919076

[24] Oldenbeuving AW, de Kort PLM, Jansen BPW, Roks G, Kappelle LJ. Delirium in Acute Stroke: A Review. Int J Stroke. 2007;2(4):270–5. 18705927 10.1111/j.1747-4949.2007.00163.x

[25] van Rijsbergen MW, Oldenbeuving AW, Nieuwenhuis-Mark RE, Nys GM, Las SG, Roks G, et al. Delirium in acute stroke: a predictor of subsequent cognitive impairment? A two-year follow-up study. J Neurol Sci. 2011;306(1–2):138–42. 21481420 10.1016/j.jns.2011.03.024

[26] Melkas S, Laurila JV, Vataja R, Oksala N, Jokinen H, Pohjasvaara T, et al. Post-stroke delirium in relation to dementia and long-term mortality. Int J Geriatr Psychiatry. 2012;27(4):401–8. 21560162 10.1002/gps.2733

[27] Sheng AZ, Shen Q, Cordato D, Zhang YY, Yin Chan DK. Delirium within Three Days of Stroke in a Cohort of Elderly Patients. J Am Geriatr Soc. 2006;54(8):1192–8. 16913984 10.1111/j.1532-5415.2006.00806.x

[28] Medeiros GC, Roy D, Kontos N, Beach SR. Post-stroke depression: A 2020 updated review. Gen Hosp Psychiatry. 2020;66:70–80. 32717644 10.1016/j.genhosppsych.2020.06.011

[29] Singh A, Black SE, Herrmann N, Leibovitch FS, Ebert PL, Lawrence J, et al. Functional and Neuroanatomic Correlations in Poststroke Depression. Stroke. 2000;31(3):637–44. 10700497 10.1161/01.str.31.3.637

[30] Rafsten L, Danielsson A, Sunnerhagen KS. Anxiety after stroke: A systematic review and meta-analysis. J Rehabil Med. 2018;50(9):769–78. 30184240 10.2340/16501977-2384

[31] Thingstad P, Askim T, Beyer MK, Bråthen G, Ellekjær H, Ihle-Hansen H, et al. The Norwegian Cognitive impairment after stroke study (Nor-COAST): study protocol of a multicentre, prospective cohort study. BMC Neurol. 2018;18(1):193. 30477436 10.1186/s12883-018-1198-x PMC6260901

[32] Reisberg B, Ferris SH, de Leon MJ, Crook T. The global deterioration scale for assessment of primary degenerative dementia. Am J Psychiatry. 1982;139(9):1136–9. 7114305 10.1176/ajp.139.9.1136

[33] Charlson ME, Pompei P, Ales KL, MacKenzie CR. A new method of classifying prognostic comorbidity in longitudinal studies: development and validation. J Chronic Dis. 1987;40(5):373–83. 3558716 10.1016/0021-9681(87)90171-8

[34] Reisberg B, Ferris SH, Kluger A, Franssen E, Wegiel J, de Leon MJ. Mild cognitive impairment (MCI): a historical perspective. Int Psychogeriatr. 2008;20(1):18–31. 18031593 10.1017/S1041610207006394

[35] Lyden PD, Lu M, Levine SR, G. BT, Broderick J. A modified National institutes of health stroke scale for use in stroke clinical trials: preliminary reliability and validity. Stroke. 2001;32(6):1310–7. 11387492 10.1161/01.str.32.6.1310

[36] Fick DM, Inouye SK, Guess J, Ngo LH, Jones RN, Saczynski JS, et al. Preliminary development of an ultrabrief two-item bedside test for delirium. J Hosp Med. 2015;10(10):645–50. 26369992 10.1002/jhm.2418 PMC4665114

[37] Hall RJ, Meagher DJ, MacLullich AMJ. Delirium detection and monitoring outside the ICU. Best Pract Res Clin Anaesthesiol. 2012;26(3):367–83. 23040287 10.1016/j.bpa.2012.07.002

[38] Marcantonio ER. Delirium in Hospitalized Older Adults. N Engl J Med. 2017;377(15):1456–66. 29020579 10.1056/NEJMcp1605501 PMC5706782

[39] Nasreddine ZS, Phillips NA, Bedirian V, Charbonneau S, Whitehead V, Collin I. The Montreal cognitive assessment, MoCA: a brief screening tool for mild cognitive impairment. J Am Geriatr Soc. 2005;53(4):695–9. 15817019 10.1111/j.1532-5415.2005.53221.x

[40] Zigmond A, Snaith R. The Hospital Anxiety and Depression Scale. Acta Psychiatr Scand. 1983;67(6):361–70. 6880820 10.1111/j.1600-0447.1983.tb09716.x

[41] Leiknes KA, Dalsbø TK, Siqveland J. Måleegenskaper ved den norske versjonen av Hospital Anxiety and Depression Scale (HADS). In: Psychometric assessment of the Norwegian version of the Hospital Anxiety and Depression Scale (HADS). Oslo: Folkehelseinstituttet; 2016.

[42] Kaufer DI, Cummings JL, Ketchel P, Smith V, MacMillan A, Shelley T, et al. Validation of the NPI-Q, a Brief Clinical Form of the Neuropsychiatric Inventory. J Neuropsychiatry Clin Neurosci. 2000;12(2):233–9. 11001602 10.1176/jnp.12.2.233

[43] Austin PC, White IR, Lee DS, van Buuren S. Missing Data in Clinical Research: A Tutorial on Multiple Imputation. Can J Cardiol. 2021;37(9):1322–31. 33276049 10.1016/j.cjca.2020.11.010 PMC8499698

[44] Lydersen S, Langaas M, Bakke Ø. The exact unconditional z-pooled test for equality of two binomial probabilities: optimal choice of the Berger and Boos confidence coefficient. Journal of Statistical Computation and Simulation. 2012;82(9):1311–6.

[45] O'Kelly M, Ratitch B. Clinical Trials with Missing Data. United Kingdom: Wiley; 2014.

[46] Munthe-Kaas R, Aam S, Ihle-Hansen H, Lydersen S, Knapskog A-B, Wyller TB, et al. Impact of different methods defining post-stroke neurocognitive disorder: The Nor-COAST study. Alzheimers Dement (N Y). 2020;6(1):e12000. 32211505 10.1002/trc2.12000 PMC7085256

[47] Goldberg TE, Chen C, Wang Y, Jung E, Swanson A, Ing C, et al. Association of Delirium With Long-term Cognitive Decline: A Meta-analysis. JAMA Neurol. 2020;77(11):1373–81. 32658246 10.1001/jamaneurol.2020.2273 PMC7358977

[48] Pendlebury ST, Rothwell PM. Prevalence, incidence, and factors associated with pre-stroke and post-stroke dementia: a systematic review and meta-analysis. Lancet Neurol. 2009;8(11):1006–18. 19782001 10.1016/S1474-4422(09)70236-4

[49] Brainin M, Tuomilehto J, Heiss WD, Bornstein NM, Bath PM, Teuschl Y. Post-stroke cognitive decline: an update and perspectives for clinical research. Eur J Neurol. 2015;22(2):229–38. 25492161 10.1111/ene.12626

[50] Fick DM, Agostini JV, Inouye SK. Delirium Superimposed on Dementia: A Systematic Review. J Am Geriatr Soc. 2002;50(10):1723–32. 12366629 10.1046/j.1532-5415.2002.50468.x

[51] Caamaño-Isorna F, Corral M, Montes-Martínez A, Takkouche B. Education and Dementia: A Meta-Analytic Study. Neuroepidemiology. 2006;26(4):226–32. 16707907 10.1159/000093378

[52] Vodermaier A, Linden W, Siu C. Screening for Emotional Distress in Cancer Patients: A Systematic Review of Assessment Instruments. JNCI. 2009;101(21):1464–88. 19826136 10.1093/jnci/djp336 PMC3298956

[53] Sagen U, Vik TG, Moum T, Mørland T, Finset A, Dammen T. Screening for anxiety and depression after stroke: Comparison of the Hospital Anxiety and Depression Scale and the Montgomery and Åsberg Depression Rating Scale. J Psychosom Res. 2009;67(4):325–32. 19773025 10.1016/j.jpsychores.2009.03.007

[54] Kowalska K, Droś J, Mazurek M, Pasińska P, Gorzkowska A, Klimkowicz-Mrowiec A. Delirium Post-Stroke: Short- and Long-Term Effect on Depression, Anxiety, Apathy and Aggression (Research Study—Part of PROPOLIS Study). J Clin Med. 2020;9(7):2232. 32674417 10.3390/jcm9072232 PMC7408940

[55] Bergersen H, Frøslie KF, Stibrant Sunnerhagen K, Schanke A-K. Anxiety, Depression, and Psychological Well-being 2 to 5 years Poststroke. J Stroke Cerebrovasc Dis. 2010;19(5):364–9. 20547073 10.1016/j.jstrokecerebrovasdis.2009.06.005

[56] Fure B, Wyller T, Engedal K, Thommessen B. Emotional symptoms in acute ischemic stroke. Int J Geriatr Psychiatry. 2006;21(4):382–7. 16534769 10.1002/gps.1482

[57] Mitchell AJ, Sheth B, Gill J, Yadegarfar M, Stubbs B, Yadegarfar M, et al. Prevalence and predictors of post-stroke mood disorders: A meta-analysis and meta-regression of depression, anxiety and adjustment disorder. Gen Hosp Psychiatry. 2017;47:48–60. 28807138 10.1016/j.genhosppsych.2017.04.001

[58] Fure B. Depresjon, angst og andre emosjonelle symptomer ved slag. Tidsskriftet for den norske legeforening. 2007;127:1387–99. 17519996

[59] MacLullich AMJ, Beaglehole A, Hall RJ, Meagher DJ. Delirium and long-term cognitive impairment. Int Rev Psychiatry. 2009;21(1):30–42. 19219711 10.1080/09540260802675031

[60] Olsson T. Activity in the Hypothalamic-Pituitary- Adrenal Axis and Delirium. Dement Geriatr Cogn Disord. 1999;10(5):345–9. 10473937 10.1159/000017168

[61] Trzepacz P, van der Mast R. The neuropathophysiology of delirium. In: Lindesay J, Rockwood K, editors. Delirium in Old Age. Oxford: Oxford University Press; 2002.

[62] Gustafson Y, Olsson T, Asplund K, Hägg E. Acute Confusional State (Delirium) Soon after Stroke is Associated with Hypercortisolism. Cerebrovasc Dis. 1993;3(1):33–8.

[63] Hashemi MM, Zhang W, Kaldewaij R, Koch SBJ, Smit A, Figner B, et al. Human defensive freezing: Associations with hair cortisol and trait anxiety. Psychoneuroendocrinology. 2021;133:105417. 34571456 10.1016/j.psyneuen.2021.105417

[64] Lovallo WR. Stress and Health. 3rd ed. United States: Sage; 2015.

[65] Joëls M. Corticosteroids and the brain. J Endocrinol. 2018;238(3):R121–r30. 29875162 10.1530/JOE-18-0226

[66] Vreeburg SA, Zitman FG, van Pelt J, DeRijk RH, Verhagen JCM, van Dyck R, et al. Salivary Cortisol Levels in Persons With and Without Different Anxiety Disorders. Psychosom Med. 2010;72(4):340–7. 20190128 10.1097/PSY.0b013e3181d2f0c8

[67] MacLullich AMJ, Deary IJ, Starr JM, Ferguson KJ, Wardlaw JM, Seckl JR. Plasma cortisol levels, brain volumes and cognition in healthy elderly men. Psychoneuroendocrinology. 2005;30(5):505–15. 15721061 10.1016/j.psyneuen.2004.12.005

[68] Otte C, Hart S, Neylan TC, Marmar CR, Yaffe K, Mohr DC. A meta-analysis of cortisol response to challenge in human aging: importance of gender. Psychoneuroendocrinology. 2005;30(1):80–91. 15358445 10.1016/j.psyneuen.2004.06.002

[69] Fjærtoft H, Skogseth-Stephani R, Indredavik B, Bjerkvik TF, Varmdal T. Norsk hjerneslagregister: Årsrapport for 2020. 2021.

[70] Ely EW, Inouye SK, Bernard GR, Gordon S, Francis J, May L, et al. Delirium in Mechanically Ventilated PatientsValidity and Reliability of the Confusion Assessment Method for the Intensive Care Unit (CAM-ICU). JAMA. 2001;286(21):2703–10. 11730446 10.1001/jama.286.21.2703

[71] Mitasova A, Kostalova M, Bednarik J, Michalcakova R, Kasparek T, Balabanova P, et al. Poststroke delirium incidence and outcomes: Validation of the Confusion Assessment Method for the Intensive Care Unit (CAM-ICU)*. Crit Care Med. 2012;40(2):484–90. 22001583 10.1097/CCM.0b013e318232da12

[72] Inouye SK. The dilemma of delirium: clinical and research controversies regarding diagnosis and evaluation of delirium in hospitalized elderly medical patients. Am J Med. 1994;97(3):278–88. 8092177 10.1016/0002-9343(94)90011-6

[73] Kuvås KR, Saltvedt I, Aam S, Thingstad P, Ellekjær H, Askim T. The Risk of Selection Bias in a Clinical Multi-Center Cohort Study. Results from the Norwegian Cognitive Impairment After Stroke (Nor-COAST) Study. Clin Epidemiol. 2020;12:1327–36. 33293871 10.2147/CLEP.S276631 PMC7718873

[74] Fjærtoft H, Indredavik B, Mørch B, Phan A, Skogseth-Stephani R, Krizak Halle K, et al. Årsrapport Norske hjerneslagsregister 2017. Trondheim: St. Olavs hospital; 2018.

[75] Kalaria RN, Akinyemi R, Ihara M. Stroke injury, cognitive impairment and vascular dementia. Biochim Biophys Acta. 2016;1862(5):915–25. 26806700 10.1016/j.bbadis.2016.01.015 PMC4827373

[76] Ilut S, Stan A, Blesneag A, Vacaras V, Vesa S, Fodoreanu L. Factors that influence the severity of post-stroke depression. J Med Life. 2017;10(3):167–71. 29075345 PMC5652262

[77] Rockwood K, Mitnitski AB, MacKnight C. Some mathematical models of frailty and their clinical implications. Rev. Clin. Gerontol. 2002;12(2):109–17.

[78] Taylor-Rowan M, Keir R, Cuthbertson G, Shaw R, Drozdowska B, Elliott E, et al. Pre-Stroke Frailty Is Independently Associated With Post-Stroke Cognition: A Cross-Sectional Study. J Int Neuropsychol Soc. 2019;25(5):501–6. 30821222 10.1017/S1355617719000092

[79] Maldonado JR. Delirium pathophysiology: An updated hypothesis of the etiology of acute brain failure. Int J Geriatr Psychiatry. 2018;33(11):1428–57. 29278283 10.1002/gps.4823

[80] Neerland BE, Watne LO, Wyller TB. Delirium in elderly patients. Tidsskr Nor Laegeforen. 2013;133(15):1596–600. 23970274 10.4045/tidsskr.12.1327

