# Correction: The relationship of acute delirium with cognitive and psychiatric symptoms after stroke: a longitudinal study

**DOI:** 10.1186/s12883-025-04055-1

**Published:** 2025-02-11

**Authors:** Vilde Nerdal, Elise Gjestad, Ingvild Saltvedt, Ragnhild Munthe‑Kaas, Hege Ihle‑Hansen, Truls Ryum, Stian Lydersen, Ramune Grambaite

**Affiliations:** 1 Department of Psychology, Norwegian University of Science and Technology, Dragvoll Bygg 12, Edvard Bulls veg 1, Trondheim, 7491 Norway; 2 Clinic of Medicine, St. Olavs Hospital, Trondheim University Hospital, Trondheim, Norway; 3 Department of Neuromedicine and Movement Science, Norwegian University of Science and Technology, Trondheim, Norway; 4 Department of Geriatrics, St. Olavs Hospital, Trondheim University Hospital, Trondheim, Norway; 5 Department of Medicine, Barum Hospital, Vestre Viken Hospital Trust, Sandvika, Norway; 6 Institute of Clinical Medicine, University of Oslo, Oslo, Norway; 7 Department of Neurology, Oslo University Hospital, Oslo, Norway; 8 Department of Medicine, Barum Hospital, Vestre Viken Hospital Trust, Sandvika, Norway; 9 Department of Mental Health, Faculty of Medicine and Health Sciences, Norwegian University of Science and Technology, Trondheim, Norway; 10 Health Services Research Unit (HOKH), Akershus University Hospital, Lorenskog, Norway

**Correction: BMC Neurol 22**,** 234 (2022)**

10.1186/s12883-022-02756-5

Following publication of the original article [1], the authors found that the following errors had occurred:

1.

In Table 2, the data for Global Deterioration scale (GDS) and for aphasia were partly incorrect. The correct data are shown in Table 2 below.

2.

On Page 3 there was a typo in the sentence.

“The Charlson Comorbidity Index [33] was used to classify the extent of comorbid diseases as mild (0–2), moderate [3–5] or severe (≥ 5), and was registered upon admission.”

The correct sentence is:

“The Charlson Comorbidity Index [33] was used to classify the extent of comorbid diseases as mild (0–2), moderate (3–5) or severe (≥ 5), and was registered upon admission.”

3.

On page 5 there was a typo in the sentence.

“Patients were hospitalized for mean 0.84 days, and 88% were admitted within day one of symptom debut.”

The correct sentence is:

“Patients were hospitalized for mean 7.53 days, and 88% were admitted within day one of symptom debut.”

**Table 2:** **(Incorrect)** Group differences in demographics and clinical characteristics for patients with and without delirium

	Delirium (*n* = 13)	Non-delirious (*n* = 126)	t/ x^2^	*p*
Age, *M* (*SD*)	79.5 (6.0)	70.6 (13.7)	4.34	0.000**
Years of education, *M* (*SD*)	12.5 (3.5)	13.9 (3.4)	-1.43	0.15
Gender, *n* female (%)	6 (46%)	62 (49%)	0.04	0.84
NIHSS at basline^1^, *M* (*SD)*	4.5 (4.6)	2.8 (3.8)	1.35	0.17
MoCA at baseline^2^, *M* (*SD*)	20.0 (2.2)	25.1 (4.7)	-3.10	0.002**
Premorbid dementia, *n* (%)	0	2	0.19	0.91
Complications^3^ > 0, n (%)	7 (54.6)	27 (21.4)	-2.15	0.05
Charlson Comorbidity Index (CCI), *M (SD)*	4.1 (1.3)	3.6 (1.9)	-1.20	0.24
Global Deterioration Scale (GDS) < 3^4^				
Pre-stroke, *n* (%)	12 (92%)	124 (98%)	0.19	0.66
3 months, *n* (%)	4 (30%)	5 (4%)	14.0	0.003**
18 months, *n* (%)	4 (31%)	11 (9%)	5.9	0.024*
36 months, *n* (%)	3 (23%)	10 (8%)	3.2	0.079
Moderate to severe aphasia^5^				
Baseline, *n* (%)	3 (23%)	19 (15%)	0.57	0.57
3 months, *n* (%)	0	4 (3%)	0.42	0.68
18 months, *n* (%)	0	3 (2%)	0.32	0.81
36 months, *n* (%)	0	0	0	1.0

*Note.*^1^Higher values indicating more severe stroke symptoms. NIHSS at baseline done at day 1 of admittance to hospital. ^2^Lower values indicating poorer global cognitive function. MoCA assessment at baseline was done either at discharge or seven days after admittance for patients with longer hospital stay. ^3^Infections, seizures, neurological progression and falls registered during hospitalization. ^4^Values < 3 indication no to very mild cognitive decline. Values > 3 indicating potential dementia. ^5^Amount of patients with a level of aphasia causing interference with conversation, indicated by the value 2 (moderate) or 3 (severe) in the NIHSS item measuring aphasia. ** indicating p-level < 0.01 * indicating p-level < 0.05

**Table 2:** **(Correct)** Group differences in demographics and clinical characteristics for patients with and without delirium

	Delirium (*n* = 13)	Non-delirious (*n* = 126)	t/ x^2^	*p*
Age, *M* (*SD*)	79.5 (6.0)	70.6 (13.7)	4.34	0.000**
Years of education, *M* (*SD*)	12.5 (3.5)	13.9 (3.4)	-1.43	0.15
Gender, *n* female (%)	6 (46%)	62 (49%)	0.04	0.84
NIHSS at baseline^1^, *M* (*SD)*	4.5 (4.6)	2.8 (3.8)	1.35	0.17
MoCA at baseline^2^, *M* (*SD*)	20.0 (2.2)	25.1 (4.7)	-3.10	0.002**
Premorbid dementia, *n* (%)	0	2	0.19	0.91
Complications^3^ > 0, *n* (%)	7 (54.6)	27 (21.4)	-2.15	0.05
Charlson Comorbidity Index (CCI), *M (SD)*	4.1 (1.3)	3.6 (1.9)	-1.20	0.24
Global Deterioration Scale (GDS) < 3^4^				
Pre-stroke, *n* (%)	12/12 (100%)	124/126 (98%)	0.19	0.92
3 months, *n* (%)	2/10 (20%)	91/120 (76%)	14.1	0.001**
18 months, *n* (%)	2/9 (22%)	71/120 (70%)	4.7	0.032*
36 months, *n* (%)	2/6 (33%)	70/84 (83%)	8.7	0.026*
Moderate to severe aphasia^5^				
Baseline, *n* (%)	2/13 (15%)	8/126 (6%)	1.44	0.27
3 months, *n* (%)	0/10	4/120 (3%)	0.34	0.80
18 months, *n* (%)	0/8	3/102 (3%)	0.24	0.89
36 months, *n* (%)	0/6	0/83	0	1.0

*Note.*^1^Higher values indicating more severe stroke symptoms. NIHSS at baseline done at day 1 of admission to hospital. ^2^Lower values indicating poorer global cognitive function. MoCA assessment at baseline was done either at discharge or seven days after admittance for patients with longer hospital stay. ^3^Infections, seizures, neurological progression and falls registered during hospitalization. ^4^Values < 3 indication no to very mild cognitive decline. Values > 3 indicating potential dementia. ^5^Amount of patients with a level of aphasia causing interference with conversation, indicated by the value 2 (moderate) or 3 (severe) in the NIHSS item measuring aphasia. ** indicating p-level < 0.01 * indicating p-level < 0.05
