# Supplementary material for: The relationship of acute delirium with cognitive and psychiatric symptoms after stroke: a longitudinal study
Source: BMC Neurol. 2022 Jun 27;22:234. doi: 10.1186/s12883-022-02756-5 (PMC9235162; doi:10.1186/s12883-022-02756-5)
Supplement: Supplementary file 1 — Additional file 1: Table S1. Details on imputation of missing values for MoCA, HADS and NPI-Q at all timepoints. [file 12883_2022_2756_MOESM1_ESM.docx]

**SUPPLEMENTARY TABLES**

**Table S1.** Details on imputation of missing values for MoCA, HADS and NPI-Q at all timepoints.

|  | Participants with no missing items | Participant with <50% missing | Participants with imputed items in analysis | >50% missing | Participants with all items missing |
| --- | --- | --- | --- | --- | --- |
| **MoCA** |  |  |  |  |  |
| 3 months | 126 | 0 | 0 of 126 | 0 | 14 |
| 18 months | 106 | 4 participants with 1 missing item | 4 of 110 | 0 | 30 |
| 36 months | 84 | 6 participants with 1 missing item | 6 of 70 | 1 participant with 19 items missing | 50 |
| **NPI-Q** |  |  |  |  |  |
| 3 months | 122 | 7 participants had 1 missing item | 7 of 129 |  | 12 |
| 18 months | 108 | 3 participants had 1 missing item | 3 of 111 | 1 participant had 8 missing items | 29 |
| 36 months | 90 | 1 participant had 1 missing item | 1 of 91 | - | 50 |
| **HADS** |  |  |  |  |  |
| 3 months | 104 | 4 participants had 1 missing item, 1 participant had 1 missing item, and 1 participant had 5 missing items. | 6 of 108 | - | 31 |
| 18 months | 101 | 1 patient with 1 missing item | 1 of 102 |  | 30 |
| 36 months | 79 | 3 patients had 1 missing item | 3 of 82 | 2 participants had 12 missing items | 59 |
